# A strategy to identify opportunities for innovation in Romanian healthcare services

**Published:** 2015

**Authors:** BI Coculescu, VL Purcărea, EC Coculescu

**Affiliations:** * “Titu Maiorescu” University, Faculty of Medicine, Bucharest, Romania; ** “Carol Davila” University of Medicine and Pharmacy, Faculty of Medicine, Bucharest, Romania; *** “Carol Davila” University of Medicine and Pharmacy, Faculty of Dental Medicine, Bucharest, Romania

**Keywords:** Romanian health system, medical management strategies, health marketing

## Abstract

In principle, the development of medicine (including the Romanian health system) is primarily dependent on the level of funding and the efficiency with which this funding is used, the structure of the population and socio-economic development of the geographical area concerned, and not least, the attitudes and expectations of patients, which in turn translate into care taking policies system.

Unlike the other services, health services are accessed by a large number of people, which results into high health. As an economic principle, the fewer resources are used to achieve the expected results, the more effective the supplier. However, the introduction of new medical technologies, many of them more reliable yet more expensive, required a reassessment of the way resources are used at the suppliers’ level to produce the expected results, an evaluation based on cost-effectiveness per analysis (or per patient) criteria. Finally, medical services are the tools of the marketing strategy of any medical organizations without which the needs and motivations of the beneficiaries (patients) could not be satisfied. In essence, the entire marketing philosophy is based on the needs and wishes of the people and on concrete solutions to solve them.

## Introduction

In countries with developed health systems, the competition among medical organizations showed that the health care system is not able to drive value for the patient, so the change is intended to become more efficient in order to achieve greater satisfaction from the patient [**1**-**3**].

Marketing is a modern, social and managerial process by which individuals and groups obtain what they need by creating, offering and exchanging products with a certain value [**4**,**5**].

The concept of marketing can be seen as a management process that, with the improvement of the methods, tactics and its instruments, give a sense of equality of products/ services in terms of quality.

The marketing idea emerged in the mid 50’s. Instead of focusing on product philosophies like “make a product and sell it”, companies have switched to one focused on the client type “intuit what the customer wants and react”, in which marketing is no longer seen as a “hunting customers” idea, but as an effort to “growing customer” and the marketers’ job is to find the right products so that business customers are able to find the right customers for no company products. The concept of marketing claims that the organization will achieve its objectives if it is shown more effective than its competitors in creating, communicating and delivering value to the customer-chosen target markets [**6**].

Marketing has emerged and evolved in close connection with the sale of physical goods, but with the growth and development, services market have come off, since 1990, marketing services becoming a distinct branch.

## Discussion

Currently, the challenge for all those involved in health care service system is how to answer the five questions of customer-oriented marketing, the time of first contact with the client/patient/health service beneficiary: ask the client what he/ she really wants; focus on what the client thinks is important; make sure you can act on feedback from your customer; get feedback on an ongoing basis; create feedback loops throughout your company.

The result of the service performance and satisfaction of users are influenced by a complex of variables. Although generally service marketing uses the same tools and techniques, performing the same functions as marketing material goods, it is characterized by some specific features, including:

• Marketing has a less important role in the decision to purchase services rather than the material goods. Thus, if the sale of goods by means of marketing consumer preference can be targeted to the respective product associated with a brand, by means of price, way of promotion and distribution, packaging etc., a hard to achieve objective for marketing services;

• Services are offered in most cases, before being rendered, a different aspect from that of material goods which are produced before their sale. There are also many types of services (including medical services) where the consumer is simultaneously the manufacturer, thus taking part in the production of the services;

• Due to the impossibility of analyzing the services provided before their execution, perception of risk by the services consumers is much higher. The assessment of services can only be done during or after the completion of their performance and consumption;

• The satisfaction of services beneficiary is greatly influenced by the way the contact staff, who act as sales personnel treats him/ her. The activity of the “frontline” staff in attitude and behavior influences the consumer’s decision to request the service organization’s services;

• The marketing system of services aims at viewing or making tangible the intangible services unlike marketing material goods, the priority being to present the tangible through abstract images;

• Promotion policy focuses on the brand of the organization providing services, and in case of material goods, the focus is on product brand;

• Sale of services involves a high degree of personalization, which aims at offering the beneficiary individualization in parallel with the establishment and strengthening of preferential long-term relationships.

**Fig. 1:**
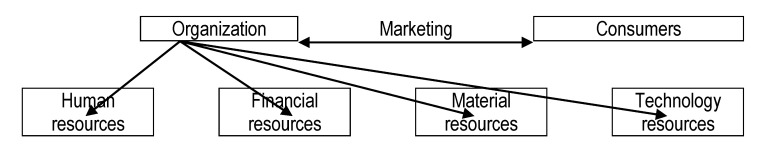
The marketing concept [**2**,**6**].

Medical services marketing is by its specificity an interdisciplinary field because it combines concepts, methods and techniques specific to both marketing services and social marketing. In addition, the patient’s needs that health services seek to meet turn this area into the boundary between the social and the economic, between orientation to benefit and non-profit.

In the context of excellence in quality health care services, meeting the healthcare systems customer’s expectations is recognized as a priority for the entire population, adopting methods and marketing concepts as an alternative to identify opportunities for innovation in health care services can only be beneficial. In this new marketing philosophy, that puts the patient in the center, all activities and policies are targeted to meet the expectations and needs of the patient, so that the quality of performance is more important than the maximum amount of benefits offered by that organization.

Some of the terms used in marketing (competition, promotion, strategy, needs, supply, price, etc.) acquire new meanings when used in defining competition between health care organizations, health promotion, medical marketing strategy, health needs, consumption of health services, the provision of care, health service charge.

Kotler and Keller define innovation as any good, service or idea perceived as new, showing that one of the key business processes is to perceive what is offered as new [**6**]. In this context, innovation in health primarily means the use of experience to develop new solutions to meet health service beneficiaries, ideas that are perceived as unique and to find enough patients willing to pay costs, therefore generating economic surplus and hence profitability. To ensure that innovation performance, a marketing strategy, is needed, what should be taken into account is the context of a innovative organizational culture in which goals are identified and target audience is defined.

Because of the long time static presentation of medical services offered, the medical unit activity stagnated and the competition eventually managed to impose on the market to the detriment of the organization. The existing habit was considered an unparalleled performance and remained preserved in the organizational culture and mentality of the health facility staff, leading to keeping the position and reputation acquired only by using accumulated resources.

## Conclusions

In healthcare, information flows freely and innovations spread quickly through contacts with patients and suppliers of equipment and drugs. Because the problems society faces can become business opportunities, interconnections that often occur unpredictably, make the emergence of new solutions and ways of development possible in the process of providing healthcare. Competition between the private and public sectors can become a managerial stimulus to overcome the inertia and routine in service delivery, with beneficial effects in adopting innovations and an innovative oriented and creative management attitude.

This change in medical organizations based on the motto “everything is marketing” develops policies and strategies based on customer needs, implies acceptance of creative thinking, knowledge institutions progressing by knowing and understanding patients to mutual trust by converting the emotions and relationships in service provision, into loyalty for a longer period of time. This implies that the organization develops a permanent bond with its patients, who are offered an added value to their request as compared to all competitors. 

“Medical Marketing interactively based on innovation” is the best method for the identifying emerging opportunities at a certain time, for stimulating consumption of health services (preventive and curative) and adopting solutions that can change in time the model of business in medical organizations.
